# Physical–Mechanical Properties and Mineral Deposition of a Pit-and-Fissure Sealant Containing Niobium–Fluoride Nanoparticles—An In Vitro Study

**DOI:** 10.3390/ma17215378

**Published:** 2024-11-04

**Authors:** Alyssa Teixeira Obeid, Tatiana Rita de Lima Nascimento, Carlos Alberto Spironelli Ramos, Rafael Francisco Lia Mondelli, Alessandra Nara de Souza Rastelli, Abdulaziz Alhotan, Marilia Mattar de Amoêdo Campos Velo, Juliana Fraga Soares Bombonatti

**Affiliations:** 1 Department of Operative Dentistry, Endodontics and Dental Materials, Bauru School of Dentistry, University of São Paulo, Alameda Octávio Pinheiro Brisolla, 9-75, Bauru 17012-901, SP, Brazil; alyssa.obeid@usp.br (A.T.O.); tatirln@gmail.com (T.R.d.L.N.); rafamond@fob.usp.br (R.F.L.M.); julianafraga@usp.br (J.F.S.B.); 2 Leibniz Institute for Solid State and Materials Research, IFW-Dresden e.V., Helmholtzstraße 20, 01069 Dresden, Germany; 3 Former Head of Endodontics Department, State University of Londrina, Londrina 86057-970, PR, Brazil; spironelli05@gmail.com; 4 Department of Restorative Dentistry, School of Dentistry, São Paulo State University—UNESP, 1680 Humaitá Street–3rd floor, Araraquara 14801-903, SP, Brazil; alessandra.nara-souza-rastelli@unesp.br; 5 Department of Dental Health, College of Applied Medical Sciences, King Saud University, P.O. Box 10219, Riyadh 12372, Saudi Arabia

**Keywords:** fluoride, nanoparticles, niobium, pit-and-fissure sealants

## Abstract

This study investigated the combined effects of adding niobium–fluoride (NbF_5_) nanoparticles to a pit-and-fissure sealant. One resin sealant was reinforced with varying amounts of nanoparticles (0.3, 0.6, and 0.9 wt%). The surface hardness (SH), energy-dispersive X-ray spectroscopy (EDX), surface roughness (Ra), color change (ΔE), and mineral deposition were assessed. Bovine enamel blocks were subjected to demineralization and pH-cycling for SH. The elemental composition and Ca/P ratio were evaluated using EDX, while the mineral deposition was measured using Fourier transform infrared spectroscopy (FTIR). Data were analyzed using ANOVA and Tukey’s test for the SH and EDX, ΔE, and Kruskal–Wallis for the Ra. The NbF_5_ modification increased the SH, with the 0.9 wt% sealant exhibiting higher SH values, and the 0.3 wt% one exhibiting significant differences compared to the control and the 0.9 wt% (*p* = 0.00) samples, even after pH-cycling. For the EDX analysis, the 0.3 and 0.6 wt% samples exhibited higher Ca/P ratios, with the 0.3% one showing evidence of P-O crystal formation. There was no significant difference in the Ra (*p* = 0.458), and the 0.6 and 0.9 wt% ones showed lower ΔE values compared to the control. The 0.3 wt% NbF_5_ demonstrated improved overall properties, making these results particularly promising for preventing tooth decay, reducing demineralization through increased ions release and promoting remineralization in posterior teeth.

## 1. Introduction

Dental caries commonly affect the occlusal pits and fissures in the posterior teeth of young adults and children because of the specific morphologies of these surfaces, including their shape, depth, and narrowness [1]. These features create environments in which biofilm can easily accumulate, impeding self-cleaning by the tongue, food, and cheeks and reducing the effectiveness of mechanical cleaning and fluoride (F) treatments [2,3]. Applying sealants to these surfaces, which can both treat and prevent early enamel caries, has been shown to be effective in preventing cavities. Sealants create a protective barrier that blocks plaque growth by limiting its access to nutrients [4].

The primary issue leading to the loss of integrity in pit-and-fissure sealants is material wear, particularly on occlusal surfaces [5,6]. Also, the effectiveness and longevity of the materials depend significantly on their ability to penetrate pits and fissures and adhere to the lateral walls. If biofilm accumulates or microleakage occurs at the material margins, bacteria from the oral environment can enter the tooth, reducing the sealant’s effectiveness and potentially leading to caries beneath the sealed area [7,8]. Mechanical tests, like microhardness, are used to evaluate wear resistance and provide a reliable measure of sealant durability. As sealants degrade over time and may require reapplication, treatment costs can be high [6,9,10]. Therefore, maintaining the integrity of pit-and-fissure sealants, which involves improving their physical and mechanical properties, is crucial for successful treatment. Additionally, preserving this interface over the long term may be essential for reducing the need for restorations due to adjacent caries [4].

Fluoride-releasing sealants positively impact tooth structure and the oral environment, but F is typically released in diminishing quantities over time and needs to be recharged [11]. Thus, the bioavailability of active F ions is essential in the dental caries process, as it inhibits demineralization and promotes remineralization. F facilitates the diffusion of calcium (Ca) and phosphate (P) ions, which help rebuild crystalline structures in carious lesions [12]. Previous studies have demonstrated that calcium–fluoride nanoparticles (nCaF_2_) incorporated into a pit-and-fissure sealant exhibit significant F release and strong antibacterial properties, showing great potential for caries inhibition [13]. So, sealants that release Ca and P ions in response to pH changes, combined with their antimicrobial and rechargeable properties, may serve as an effective complementary strategy for managing dental caries [14]. However, challenges remain, particularly due to the weak chemical bonds among the CaP particles within the resin matrix [15], which often fails to provide adequate mechanical properties. The main challenge is the development of materials that effectively balance remineralization with strong mechanical properties to prevent flaws and cracks [16]. Integrating bioactive components with improved mechanical properties into sealants may be desirable to transform them into a more dynamic product, facilitating mineral nucleation near demineralized areas, allowing for deeper penetration and remineralization of enamel layers, and providing additional ions to repair subsurface lesions for a longer period [17,18].

Commonly studied fillers, such as bioactive glasses [3,5,19] and various oxides [16,20], like niobium (Nb) fillers, have been used to enhance resin-based materials by providing added bioactivity [21,22,23]. When associated with copper, surface mineral deposition was observed in a one-step bonding agent, resulting in a more homogeneous and uniform tooth/restoration interface [23]. Nb oxide has garnered researchers’ attention due to its notable physicochemical properties as a semiconductor oxide with high energy absorption, excellent biocompatibility, strong potential in biomedical applications, and optical properties similar to natural tooth structures [21,24,25]. Additionally, it promotes the growth of hydroxyapatite crystals when in contact with human saliva [26].

It is valuable to investigate the combined and synergistic effects of incorporating both F and Nb into a pit-and-fissure sealant. Modifying resin sealants with NbF_5_ nanoparticles could enhance the material’s resistance to cariogenic challenges and prolong its effectiveness by providing bioactive and remineralizing properties, which are crucial for effective protection and reducing the need for replacements. Sustained F release is particularly beneficial for high-risk cavity patients [27], and it is important to achieve adequate F release while keeping the filler content low [28]. Such advanced materials could represent a significant advancement in modern caries management strategies. However, modifying a material can affect its characteristics [20]. Therefore, this study aimed to assess the mechanical properties and mineral deposition of a pit-and-fissure sealant incorporated with NbF_5_ nanoparticles at varying concentrations (0.3 wt%, 0.6 wt%, and 0.9 wt%). The null hypothesis was that incorporating NbF_5_ nanoparticles into the sealant would not result in differences in the mechanical properties or mineral deposition of the sealant compared to the unmodified version.

## 2. Materials and Methods

### 2.1. Experimental Design

A pit-and-fissure sealant composed of TEGDMA, BisGMA, and UDMA (PacSeal, Pac-Dent International, Brea, CA, USA) was used as the control material. This material was then modified with different concentrations of NbF_5_ nanoparticles. The NbF_5_ nanoparticles had a formula weight of 187.9 g/mol and were purchased from Sigma Aldrich, Saint Louis, MO, USA. The gravimetric analysis (% Nb after ignition to oxide) ranged from 48.2% to 50.7%, confirming the presence of Nb after analysis. Additionally, the nanoparticles appeared as a powder, with colors ranging from white to off-white [29].

The surface hardness (SH) (*n* = 10), energy-dispersive X-ray spectroscopy (EDX), surface roughness (Ra; µm) (*n* = 7), color change (ΔE) (*n* = 7), and mineral deposition by Fourier transform infrared spectroscopy (FTIR) (*n* = 3) of all groups were measured.

### 2.2. Preparation and Selection of Enamel Specimens and Caries-like Lesion Evidence

Forty extracted, non-carious upper central incisors from bovine teeth were obtained under a protocol registered and approved by the Animal Use Ethics Committee (#012/2024).

The teeth were selected using a 10^x^ magnifying glass for support. Any specimens that exhibited cracks, caries, and/or fractures were excluded. The roots of each tooth were sectioned 1 mm below the enamel–cementum junction using an IsoMet low-speed saw (Buehler; Lake Bluff, IL, USA) equipped with a diamond disc (Extec; Enfield, CT, USA). Subsequently, rectangular enamel slabs were prepared (6 × 4 × 2 mm^2^) at a speed of 300 rpm under continuous water irrigation. The specimens were then polished flat using abrasive papers (#600 grit to #1200 grit), followed by a felt disk with a diamond suspension (Buehler; Lake Bluff, IL, USA). The baseline SH of the dental slabs was measured using three indentations on specimens that would not receive the sealant, ensuring no interference with treatments regarding mineral loss or gain. The SH was assessed with a Knoop diamond indenter, spaced 100 μm apart, using a 50 g load for 10 s (MicroMet 6040; South Bay Technology, Lake Bluff, IL, USA). The specimens were then randomized (Excel 15.0, Microsoft, Redmond, WA, USA) into the following four treatment groups (*n* = 10 each): Control, 0.3 wt% NbF_5_ (0.3Nb/F), 0.6 wt% NbF_5_ (0.6Nb/F), and 0.9 wt% NbF_5_ (0.9Nb/F).

Before initiating the process to create simulated caries-like lesions, the lateral surfaces of each specimen were coated with an acid-resistant varnish. A circular piece of contact paper (2 mm in diameter) was then placed over the central surface of the enamel slabs. This area was also coated with the acid-resistant varnish, and once dried, the contact paper was removed, exposing a surface area of 3.14 mm^2^ to the treatments. The demineralizing solution used had the following composition: 1.3 mM Ca(NO_3_)_2_.4H_2_0; 0.78 mM Na_2_HPO_4_.2H_2_0; 0.05 M glacial acetic acid; and 0.0315 ppm fluoride, with a pH of 5.0 at 37 °C [30]. The selected specimens were immersed in this solution for 16 h (30 mL per specimen).

After the caries-like induction, the SH of the dental slabs was re-evaluated through three indentations, and transversal microradiography (TMR) was performed to confirm the creation of the artificial caries-like subsurface lesions without surface erosion in the enamel blocks. The sample size was calculated using SigmaPlot 12.0 (Systat Software, San Jose, CA, USA), with a significance level of 0.05 and 80% power.

### 2.3. Treatment of Bovine Enamel Blocks with Resin Sealant Doped with NbF_5_

The resin sealant used in this study, produced by Pac-Dent International (Brea, CA, USA), is a light-cured and glass-filled resin. Before application, all specimens were cleaned using a Robinson brush with pumice, and a thin layer of sealant material was applied using a micro brush, according to the assigned treatment groups. A portion of the resin sealant was dispensed into a container and weighed on a precision scale (Denver Instrument; Sao Paulo, Brazil) to prepare the sealant mixtures. NbF_5_ fillers were then weighed to achieve concentrations of 0.3 wt%, 0.6 wt%, and 0.9 wt% corresponding to the mass of the sealant. These fillers were incorporated into the pit-and-fissure sealant by slow, manual mixing, and the mixture was homogenized for one minute.

For all groups, the enamel substrate was etched with 38% phosphoric acid (PacEtch, Pac-Dent International, Brea, CA, USA) for 30 s, rinsed with water for 20 s, and dried using compressed air. The sealant material was then applied according to the group assignment (0 wt%, 0.3 wt%, 0.6 wt%, and 0.9 wt%) and photoactivated for 20 s using a wide-angle LED device spectrum with 1000 mW/cm^2^ irradiance (VALO Cordless, Ultradent Products Inc., South Jordan, UT, USA).

### 2.4. pH-Cycling Regimen

After the treatments, the specimens were subjected to daily immersions in 30 mL of demineralizing solution (2.0 mM Ca(NO_3_)_2_.4H_2_O, 2.0 mM NaH_2_PO_4_.2H_2_O, 0.077 mM acetate buffer, 0.02 ppm fluoride, and pH 4.7) for 6 h. Following this, they were immersed in a remineralizing solution (1.5 mM Ca(NO_3_)2.4H_2_O, 0.9 mM NaH_2_PO_4_.2H_2_O, 150 mM KCl, 0.1 mol/L buffer, and 0.03 ppm fluoride, at pH 7.0) for 18 h. This cycle was repeated for seven days at 37 °C to simulate a clinical condition in a dynamic pH-cycling regimen. During the final two days, the specimens were kept only in the remineralizing solution [31].

### 2.5. Surface Hardness (SH)

The SH was determined at the end of each time condition (after treatments and after the pH-cycling regimen). Three indentations were performed at a standard distance from the treatment area (100 μm). The mean values of the three indentations were calculated.

### 2.6. EDX Analysis

A total of four groups were evaluated under different conditions, as follows: control after pH-cycling, 0.3Nb/F after pH-cycling, 0.6Nb/F after pH-cycling, and 0.9Nb/F after pH-cycling. The specimens were sputter-coated with a thin layer of gold and examined using scanning electron microscopy (SEM) (Aspex Express; Fei Europe, Eindhoven, The Netherlands) at an accelerating voltage of 15–20 kV in a relative vacuum. Compositional information was analyzed for each group via EDX, fully integrated into the Aspex Express SEM across the entire area to determine the mass percentages of Ca and P. This analysis was conducted in the standardless mode, and the Ca/P ratio was calculated for all groups.

### 2.7. Mineral Deposition

To develop the specimens for this test, a unique ring matrix with a 2 mm height was used to hold the sealant specimen. This matrix consisted of an outer metallic ring and an inner split Teflon mold. Sealant specimens made in this mold were disc-shaped, 2 mm thick, and 2 mm in diameter. The mold was placed over the first layer of the celluloid tape with a thin glass slide underneath and then filled with sealant according to the tested groups. A second layer of celluloid tape, along with a 1 mm thick glass slide, was placed over the Teflon mold that had been filled with the testing material. The Valo light tip was centered on the specimen and light-cured (1000 mW/cm^2^ for 40 s; VALO; Ultradent, UT, USA) [32].

Specimens from each group (control, 0.3Nb/F, 0.6Nb/F, and 0.9Nb/F) were immersed in 5 mL of the described solution (sodium chloride (NaCl), potassium chloride (KCL), di-potassium hydrogen phosphate trihydrate (K_2_HPO_4_.3H_2_O), magnesium chloride hexahydrate (MgCl_2_.6H_2_O), calcium chloride (CaCl_2_), sodium sulfate (Na_2_SO_4_), tris-hydroxymethyl aminomethane (Tris) buffer, and 1 M hydrochloric acid, with the pH adjusted to 7.4). The specimens were maintained in airtight containers in a 37 °C oven for the designated times, as follows: T0—initial time, T14—14 days after specimen immersion, and T21—21 days after specimen immersion. The resin sealants were subjected to FTIR (Shimadzu Corporation, Model IR Prestige 21, Kyoto, Japan) analysis at T0, T14, and T21.

### 2.8. Surface Roughness (Ra, µm)

The Ra of all groups was assessed after the polymerization of specimens (13 × 1 mm^2^) using a roughness tester (Hommel Tester RT 1000, Hommelwerke, GmbH, Alte Tuttinger Strebe 20. D-7730 VS-Schwenningen, Germany), with a 0.80 mm cutoff, 0.0001 μm resolution (8 μm range), 0.5 mm/s speed, and a total length of 4 mm. A diamond needle was used to perform five surface readings in random directions, and the mean Ra was calculated. The reading parameters were determined with an assessment length (Lt) of 5 mm and a cutoff length (Lc) of 0.25 mm.

### 2.9. Color Change (ΔE)

For the ΔE test, the color was assessed at different time points using a CIELab-based colorimeter (Vita EasyShade-Vita Zahnfabrik, Bad Sackingen, Germany) on specimens (4 × 4 mm^2^). Before the measurements, the spectrophotometer was calibrated following the manufacturer’s instructions. An initial measurement (P0) was taken 24 h after the specimen’s fabrication, a second measurement (P1) was taken 7 days after P0, and a third measurement (P2) was taken after artificial aging, which involved 24 h of water storage at 60 °C [33]. Between P0 and P1, all specimens were stored in dry conditions at 37 °C without exposure to light. Three consecutive measurements were made in the center of each specimen until uniform values were observed [34].

The ΔE was calculated using the following equation: ΔE = √((ΔL*)^2^ + (Δa*)^2^ + (Δb*)^2^), where ΔL*, Δa*, and Δb* represent the color differences observed between the baseline (P0) and the subsequent storage periods (P1 and P2).

### 2.10. Statistical Analysis

The SigmaPlot software, Version 12.0, was used for statistical analysis of the data. The homogeneity of variances and normal distribution of data were checked using the Levene and Shapiro–Wilk tests (*p* > 0.05), respectively. Based on these results, and with both assumptions confirmed, a parametric statistical analysis was selected. The SH and ΔE were analyzed using two-way ANOVA, while the Ca/P ratio via EDX was evaluated using one-way ANOVA followed by Tukey’s HSD test. The significance level was set at α = 0.05. For the Ra, since the data did not follow a normal distribution (*p* < 0.05), a non-parametric test, Kruskal–Wallis, was used. As for the mineral deposition test, the data were collected qualitatively, and statistical analysis was not required.

## 3. Results

### 3.1. Mechanical Properties

**Figure 1:**
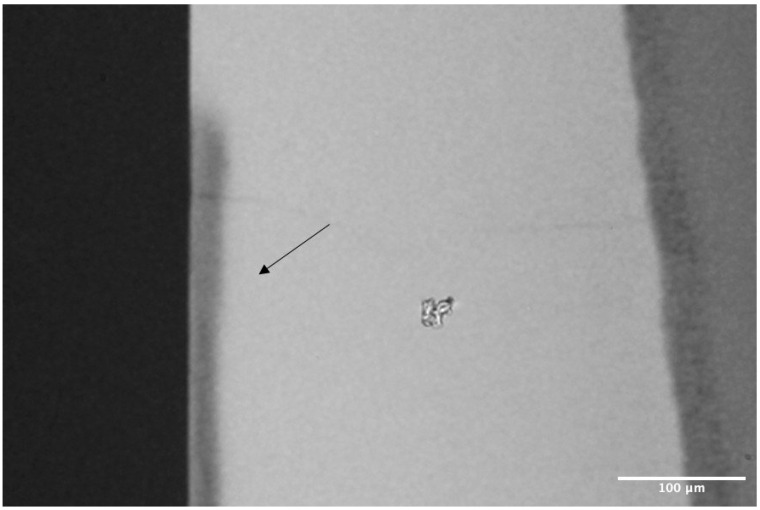
Representative TMR image (20×) of the artificial carious lesion created after immersion in demineralizing solution. The black arrow indicates the subsurface lesion.

Figure 1 presents a TMR image demonstrating the production of artificial caries-like subsurface lesions in the enamel blocks, showing no surface erosion after exposure to the demineralizing solution. For SH, the power of the test conducted with an alpha of 0.05 was 0.528. The effect size was calculated using Eta-squared, with the sample size accounting for 39% of the total variance in the SH [17]. For EDX, the power of the test conducted with an alpha of 0.05 was 1.00. The effect size was also calculated using Eta-squared, with the sample size effect accounting for 79% [17]. To determine the sample size for the ΔE and Ra, alpha and beta errors of 5% and 20% were set, respectively, using a standard deviation of 1.5 obtained from the pilot studies.

Table 1 summarizes the SH results (*n* = 10). There was no significant difference between the groups in the initial condition (Initial SH). However, after the caries, all groups exhibited a significant decrease in SH compared to the initial condition (*p* < 0.05). The sealant treatments significantly increased the hardness compared to the demineralized condition. The sealants modified with NbF_5_ nanoparticles showed a significantly greater hardness than the control group, with no significant difference between the 0.3Nb/F and 0.6Nb/F groups. The 0.9Nb/F group displayed the highest SH values (309.7 ± 2.5), which were statistically significant.

These results remained consistent after the pH-cycling regimen, with the 0.3Nb/F group showing a significant difference from the control and the 0.6Nb/F and 0.9Nb/F groups displaying higher SH values (305.9 ± 1.4 and 322.2 ± 3.4, respectively), with no significant difference among them. After the pH-cycling, the SH values of the 0.9Nb/F group were similar to those of the initial enamel condition.

**Figure 2:**
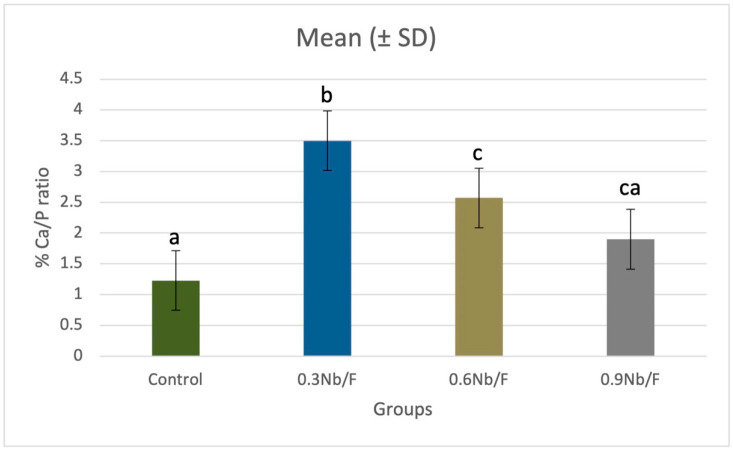
Ca/P (%) ratio calculated by EDX analysis. Different lowercase letters indicate statistically significant differences between the groups.

Table 2 presents the complete EDX elemental analysis for each group. The Ca/P ratio values for all groups values are presented in Figure 2. The 0.3Nb/F group exhibited the highest ratio (3.5%), which was significantly different compared to the 0.6Nb/F (2.5%) (*p* = 0.012), the 0.9Nb/F (1.9%) (*p* = 0.004) and the control group (1.2%) (*p* = 0.001). There was no statistical difference between the control group and the 0.9Nb/F group (*p* = 0.141), nor between the 0.9Nb/F and 0.6Nb/F groups (*p* = 0.331).

Regarding the Ra (*n* = 7), no significant differences were found for all groups (*p* = 0.458), and the power of the performed test (0.049) was below the desired power of 0.800 (Table 3). For the ΔE (*n* = 7), the initial mean results across all groups showed no statistical differences (*p* = 0.186). After water storage at 60 °C, the 0.3Nb/F group did not significantly differ from the control group (*p* = 0.131). However, the 0.6Nb/F and 0.9Nb/F groups exhibited the lowest ΔE values, at 5.8 ± 1.4 and 5.9 ± 1.3, respectively, indicating lower differences in the ΔE compared to the control group (8.7 ± 1.9), with statistically significant differences (*p* < 0.05) (Table 3).

### 3.2. Mineral Deposition

**Figure 3:**
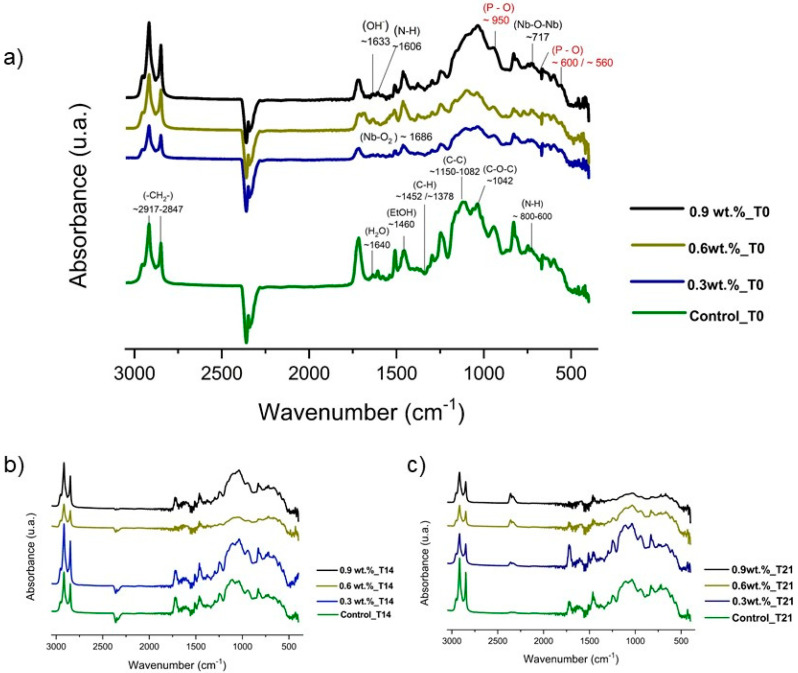
FTIR spectra of the specimens according to the studied time points: (**a**) T0; (**b**) T14; (**c**) T21. The red highlight indicates the peaks of the PO_4_^3−^ groups on the sample’s surface.

Figure 3a–c display the spectra of specimens for all test materials at different time points, as follows: (3a) T0—initial time; (3b) T14—14 days after the specimen’s immersion; and (3c) T21—21 days after the specimen’s immersion. In all spectra, no deformation bands of the composite were observed in the region of ~3425–3193 cm^−1^ (axial deformation), which may indicate the uniformity and homogeneity of the composite. The spectrum of the sealant can be observed at time T0 in Figure 3a. The region around ~1606 cm^−1^ shows evidence of angular deformation, and at 800–664 cm^−1^, symmetric out-of-plane angular deformations attributed to the (N-H) bond of the amide group related to the residual solvents containing amide. Silica peaks (Si-O-Si) were reported at ~790 cm^−1^, which overlapped with the (-OH) peaks. Bands related to the (-CH_2_) group were observed at 1378 cm^−1^. The bands at 2917–2847 cm^−1^ refer to asymmetric and symmetric stretching of this same group. The regions between 1150 and 1082 cm^−1^ (symmetric and asymmetric stretching) and ~490 and 450 cm^−1^ (axial deformation) refer to the vibrational modes of the (C-C) bond. Additionally, the bonding band (Nb-O) was observed at ~1626 cm^−1^ and the group (Nb-O-Nb) at ~721 cm^−1^. Notably, the (Nb-F or Nb-C) bands were not detected in these spectra because of the sensitivity of the assay. In all spectra, the band in the region of ~2917 to 2847 cm^−1^ was attributed to methylene carbon. On the same spectrum, bands were also observed at 1633 cm^−1^, referring to hydroxyl, and at ~1640 cm^−1^, referring to residual water or prominent moisture on the surface of the samples that were soaked in the bioactive solution. Additionally, ethanol residue was observed in the region of ~1460 cm^−1^ to 1420 cm^−1^, with the peak referring to the (C-O) group adjacent to the carbonyl, and at ~1042 cm^−1^, which can be attributed to the (C-O-C) group adjacent to the ester group of the sealing compound [35].

The FTIR analysis revealed the formation of a characteristic halo of free P (PO^4−^) band nucleation on the surfaces of the resin sealants across all groups. Peaks were observed in the range of approximately 500 to 1000 cm^−1^ at time points T0, T14, and T21. At 14 days (Figure 3b), peaks at ~560 cm^−1^, ~600 cm^−1^, and ~950 cm^−1^ indicated the initiation of P-O crystal formation. This suggests the deposition of apatite layers on the surface of the material, indicating a potential bioactive function in the resin sealants. A chemical bioactivity test can provide evidence of P deposition, and FTIR spectra can suggest the presence of bonds and groups on the material’s surface. However, peak overlap or equivalence may occur, which is better explored through microscopy analyses such as EDX [36,37].

## 4. Discussion

In preventive dentistry, pit-and-fissure sealants have been used to effectively prevent caries in permanent molars, demonstrating positive outcomes [38]. This study evaluated experimental pit-and-fissure sealants containing NbF_5_ nanoparticles for their physical–mechanical properties and mineral deposition potential. Based on study’s findings, the initial hypothesis was rejected.

Dental caries are a widespread disease, and pit-and-fissure sealant therapy is an ultraconservative approach used to protect and seal pits and fissures on occlusal tooth surfaces [39]. Various operative factors, including moisture control, surface preparation, and the application of bonding agents, affect the clinical outcomes. Furthermore, the sealant material is continually affected by intraoral fluids and temperature fluctuations, which can lead to microleakage and compromise performance [40,41]. Among the materials used, resin sealants are preferred because of their superior retention and effective caries preventive, while glass ionomer is recommended for situations in which moisture control is difficult [42].

Nanotechnology has transformed dentistry by enabling the development of advanced dental materials and facilitating the creation of smart materials. In this way, nanoparticles are integrated into dental materials for various purposes, including providing antibacterial effects, enhancing the flexural strength of composites as fillers, coating implant surfaces to improve osseointegration, and serving as local drug delivery systems for therapeutic effects [43,44,45]. Inorganic nanoparticles, such as metal oxides, like copper, silver, silica, zinc oxide, titanium oxide, and zirconium oxide, are the most widely used in dental materials. These nanoparticles can enhance the mechanical properties of materials, provide antimicrobial effects, and improve biocompatibility [46]. Nb on the other hand, has shown promise in the biomedical field due to its exceptional physical, chemical, and biological properties, although it is still relatively underexplored in dentistry [21,25].

Adding F into the resin sealant’s composition can significantly reduce bacterial adhesion to tooth surfaces [47], increase the enamel’s SH [48], and arrest the progression of caries [49]. These benefits are due to a reduction in demineralization and an improvement in remineralization, offering protection to adjacent teeth even after the material has worn away on occlusal surfaces [50,51]. However, the release of ions is transient [11]. To the best of the author’s knowledge, no in vitro studies have yet been published demonstrating the efficacy of the combining F and Nb in a pit-and-fissure sealant.

Regarding the cost implications, preventive care is more sustainable and less costly compared to restorative and surgical treatments [38]. Sealants containing F offer a noninvasive prevention option associated with decreased caries incidence, increased time to caries development, and greater cost-effectiveness [52]. Additionally, Nb oxide has emerged as a potentially effective photocatalyst due to its good UV light absorption, stability, and low cost [53]. Overall, the total Nb market value was approximately USD 1389 million in 2019, and it is anticipated to reach USD 1748 million by the end of 2027 [54]. Brazil has the largest Nb reserves, accounting for about 98.53% of the total Nb in the world. Ore reserves in Brazil exceed 212 billion tons, making it the largest producer of Nb, contributing around 96% of global production. Canada and Australia follow Brazil, with 1.01% and 0.46% of the world’s Nb ore reserves, respectively [55,56]. Therefore, incorporating nanoparticles with bioactive potential could reduce treatment costs.

Sealants are subjected to wear and fracture under compressive or flexural stresses [57] due to complex masticatory forces during chewing. This makes it critical to assess their SH to ensure they can withstand wear and occlusal forces, thereby extending their durability [13,58]. This property is a crucial factor in dental materials science, as it may serve as an indicator of a material’s mechanical resilience by evaluating its performance, particularly in terms of its ability to inhibit enamel demineralization and enhance hardness during pH-cycling challenges [17]. The results of this study suggest that as the concentration of nanoparticles increases, the material becomes more resistant to wear and more effective at inhibiting demineralization, even after pH-cycling. However, all groups exhibited remineralization potential after treatment with sealants containing NbF_5_ nanoparticles, by comparing the difference between groups after demineralization and treatment processes. The 0.9Nb/F group displayed higher SH values, indicating improved remineralization of caries-like lesions. These results remained consistent after exposure to a pH-cycling regimen, with the 0.6Nb/F and 0.9Nb/F groups showing increased values, and 0.3Nb/F exhibiting no significant difference compared to the control group. After the pH-cycling, the 0.9Nb/F group showed SH values similar to those of the initial enamel condition. Despite the incorporation of nanoparticles, the viscosity of the materials likely remained unchanged, as filler concentrations in low-viscosity dental materials must kept below 15 wt% to avoid affecting penetration and viscosity [59]. Although the degree of conversion will be evaluated in future studies, a lower filler content might improve the distribution of reactive groups, leading to higher degree of polymerization [60].

The EDX test is used to measure the mineral content of materials at an ultrastructural level, facilitating the assessment of the remineralization capacity of various substances [61,62]. In this study, enamel remineralization was evaluated by analyzing specimens using EDX to determine the mass percentages of key minerals, including Ca and P, which are the primary components of hydroxyapatite deposited on a material’s surface. The Ca/P ratio was utilized to evaluate the effects of NbF_5_ nanoparticles on enamel remineralization. This ratio was calculated for all groups, with the 0.3Nb/F and 0.6Nb/F groups exhibiting higher values compared to the 0.9Nb/F and control groups. The lowest Ca/P ratios were observed for the control and 0.9Nb/F groups. The 0.3Nb/F and 0.6Nb/F groups demonstrated bioactive potential without compromising the surface roughness (Ra), which may influence biofilm accumulation and reduce the risk of recurrent carious lesions.

Moreover, the sealant modified with 0.3Nb/F released significantly more F ions than the control, enhancing the resistance of the tooth structure, preventing demineralization, and promoting the remineralization of dental lesions. The Si-O and F-compounds present in the control group were already capable of capturing P and Ca ions because of their reactivity and instability at high concentrations. However, the uncontrolled agglomeration of Si nanoparticles or their excessive presence on the material’s surface may hinder successful interaction with Ca [63]. In contrast, NbF_5_ can chemically balance and stabilize free Ca ions. Thus, our study demonstrated that a sealant doped with the Nb-F compound, even at a low concentration of 0.3 wt%, can enhance the capture of P and Ca ions. Over time, this can increase the crystallization of Ca and P compounds, promoting a thick layer on the surface and preventing the invasion of undesirable bacteria in the oral cavity.

Nanoparticles in dental materials can improve optical properties by enhancing light transmittance due to their smaller size compared to the wavelength of visible light [64]. The color stability of restorations is crucial for their functional lifespan, as their durability depends on the ability to retain color over many years [65]. Variations in the particle shape and size contribute to differing whiteness indices [66]. Additionally, the metal oxides present in bioceramics can affect the color stability of the material due to variations in their chemical compositions [67]. In this study, increasing the concentration of nanoparticles resulted in slight color alterations, even after aging. These changes may reflect the degree of conversion, initiator system, particle size, monomer conversion, pigment stability, and water sorption stability [34]. This suggests a lower probability of requiring a replacement and a reduced risk of restoration failure.

This study assessed mineral deposition through FTIR to assess the vibrational modes of silicate, carbonate, hydroxyl, and P, comparing different time points (T0, T14, and T21). Additionally, FTIR can simultaneously determine the effectiveness of the remineralization and the extent of the remineralization process [68]. Simulated body fluid (SBF) was used to create a supersaturated environment around the substrate [18], mimicking biomimetic mineralization by providing temperature, ion concentration, and pH conditions similar to those of human blood plasma. This environment leads to the formation of an apatite layer, which provides an additional barrier that prevents the dissolution of metal ions [69,70]. This occurs because bioactive materials chemically react with body fluids in the context of tissue repair. Consequently, the material’s ability to induce apatite formation is closely related to its biointeractivity and bioactivity, resulting in the capacity to form apatite on its surface within a short induction time [71].

While P crystals formed slowly, infrared spectrum sensitivity may not have been sufficient to detect these crystal formation bands. The bands at 460, 550–600, 960, and 1020–1120 cm^−1^ are characteristic of P groups and correspond to the asymmetric deformation of O-P-O in PO_4_^2−^. At 14 days in the 0.3Nb/F group, peaks at around 560, 600, and 950 cm^−1^ appeared, indicating the onset of P-O crystal formation, which could suggest the deposition of apatite layers on the surface of the material [72]. This finding was supported by the EDX results, which showed a higher Ca/P ratio in that group. In this study, the FTIR analysis revealed a halo characteristic of free P (PO^4−^) bands nucleating on the surface area. By 21 days, the 0.3Nb/F group resembled the control group, suggesting SBF saturation.

Materials containing reactive fillers that protect teeth from adjacent caries have emerged as part of the effort to develop bioactive composites. These materials help prevent microleakage, which is essential for effectively halting the onset and progression of caries [73]. It is important to recognize that a sealant with the improved characteristics discussed can likely protect both the fissure’s penetration area and the adjacent demineralized enamel from recurrent caries over time. Consequently, this approach could reduce the need for multiple patient visits to the dental office.

Although the 0.9Nb/F group exhibited the highest SH, the 0.3Nb/F group demonstrated favorable outcomes in several aspects after pH-cycling, indicating SH, Ra, and ΔE values similar to the control group, a higher %Ca/P ratio, and significant mineral deposition after 14 days. This investigation was an in vitro study, which may result in findings that are less reliable compared to those from studies involving human teeth and in vivo experiments. Although the lesions in bovine teeth were likely not as deep as those in human teeth, a TMR test was conducted to confirm the formation of subsurface lesions. Also, an SBF solution and pH-cycling regimen were used to simulate a long-term challenge and replicate a clinical scenario that occurs in the oral cavity every day by saliva, characterized by episodes of demineralization and remineralization of the enamel. However, it can be speculated that the low concentration of NbF_5_ nanoparticles did not compromise the tested mechanical properties, aligning with the literature that suggests the appropriate fraction of bioactive fillers in resin composites can promote remineralization through ion release [74], as long as they are well-dispersed and non-agglomerated [75]. Further studies should include a fluoride-based sealant as a positive control and incorporate SEM to evaluate P crystal layers, reinforcing the findings from the energy bands associated with P and carbonate formation during mineral deposition tests. Additionally, investigations using human enamel samples and clinical trials should be conducted to better understand how sealants behave under conditions that are closer to clinical settings. Also, the power of the performed test for Ra was below the desired power of 0.800, indicating the necessity of further studies with a larger number of specimens to verify with more accuracy whether there is no statistical difference among the groups evaluated.

## 5. Conclusions

NbF_5_ nanoparticles may be incorporated into pit-and-fissure sealants without compromising their physical–mechanical properties. The sealant doped with 0.3Nb/F nanoparticles demonstrated a more positive set of results, including signs of mineral deposition, increased SH after pH-cycling challenges, and ΔE and Ra values similar to the control group. However, further in vitro and in vivo studies are needed to investigate their chemical–physical properties in greater depth and confirm their ability to protect enamel through bioactive processes.

## Figures and Tables

**Table 1:** Surface hardness (SH) results.

Groups	Initial SH (Kg/mm^2^)	SH (Kg/mm^2^) (After Demineralization–Caries Lesion)	SH (Kg/mm^2^) (After Sealant Treatment)	SH (Kg/mm^2^) (After pH-Cycling)
Control	334.1 ± 24.3 ^Aa^	206.3 ± 1.6 ^Ab^	237.8 ± 7.5 ^Ac^	256.7 ± 4.0 ^Ac^
0.3Nb/F	320.5 ± 28.7 ^Aa^	208.0 ± 2.9 ^Ab^	281.4 ± 8.3 ^Bc^	290.5 ± 3.6 ^Bc^
0.6Nb/F	316.6 ± 23.8 ^Aa^	206.4 ± 2.3 ^Ab^	286.6 ± 7.0 ^Bc^	305.9 ± 1.4 ^BCc^
0.9Nb/F	336.3 ± 14.7 ^Aa^	208.5 ± 2.8 ^Ab^	309.7 ± 2.5 ^Cc^	322.2 ± 3.4 ^Cac^

Different uppercase letters indicate statistically significant differences between the groups for each time evaluated—vertical (*p* < 0.05); different lowercase letters indicate statistically significant differences between the times for each group evaluated—horizontal (*p* < 0.05).

**Table 2:** EDX analysis showing the complete elemental composition for each group. The mass percentages for Ca and P are highlighted in blue and red, respectively.

Group/Mass (%)	C	O	F	Mg	Al	Si	P	K	Ca	Ti	Ni
Control	23.0	35.2	6.1	0.1	5.9	16.7	0.7	0.0	0.9	4.9	6.1
0.3Nb/F	26.3	35.5	4.3	0.3	4.7	16.8	0.4	0.0	1.6	5.2	4.5
0.6Nb/F	22.7	31.5	6.7	0.4	6.8	16.1	0.6	0.2	1.6	4.9	8.8
0.9Nb/F	22.6	35.3	5.9	0.2	5.6	16.5	0.6	0.1	1.13	5.2	6.4

**Table 3:** Means ± standard deviation of the degree of color change (ΔE) and medians of the surface roughness (Ra, µm).

Groups	Degree of Color Change (ΔE) (Initial: P0–P1)	Degree of Color Change (ΔE) (Final: P0–P2)	Surface Roughness (Ra)
Control	7.2 ± 1.5 ^a^	8.7 ± 1.9 ^a^	0.033 ^a^
0.3Nb/F	7.2 ± 1.7 ^a^	7.3 ± 1.3 ^a^	0.037 ^a^
0.6Nb/F	6.9 ± 1.9 ^a^	5.8 ± 1.4 ^b^	0.042 ^a^
0.9Nb/F	7.8 ± 1.0 ^a^	5.9 ± 1.3 ^b^	0.035 ^a^

Different lowercase letters in the same column indicate statistically significant differences between the groups (*p* < 0.05).

## Data Availability

The original contributions presented in the study are included in the article, further inquiries can be directed to the corresponding author.
